# The role of positive selection in determining the molecular cause of species differences in disease

**DOI:** 10.1186/1471-2148-8-273

**Published:** 2008-10-06

**Authors:** Jessica J Vamathevan, Samiul Hasan, Richard D Emes, Heather Amrine-Madsen, Dilip Rajagopalan, Simon D Topp, Vinod Kumar, Michael Word, Mark D Simmons, Steven M Foord, Philippe Sanseau, Ziheng Yang, Joanna D Holbrook

**Affiliations:** 1 Department of Biology, University College London, Darwin Bldg, Gower Street, London WC1E 6BT, UK; 2 Computational Biology Division, Molecular Discovery Research, GlaxoSmithKline R&D Ltd., 1250 South Collegeville Road, Collegeville, PA 19426, USA; 3 Institute for Science and Technology in Medicine, Keele University, Thornburrow Drive, Hartshill, Stoke-on-Trent, ST4 7QB, UK; 4 Molecular Discovery Research Information Technology, GlaxoSmithKline R&D Ltd., 1250 South Collegeville Road, Collegeville, PA 19426, USA

## Abstract

**Background:**

Related species, such as humans and chimpanzees, often experience the same disease with varying degrees of pathology, as seen in the cases of Alzheimer's disease, or differing symptomatology as in AIDS. Furthermore, certain diseases such as schizophrenia, epithelial cancers and autoimmune disorders are far more frequent in humans than in other species for reasons not associated with lifestyle. Genes that have undergone positive selection during species evolution are indicative of functional adaptations that drive species differences. Thus we investigate whether biomedical disease differences between species can be attributed to positively selected genes.

**Results:**

We identified genes that putatively underwent positive selection during the evolution of humans and four mammals which are often used to model human diseases (mouse, rat, chimpanzee and dog). We show that genes predicted to have been subject to positive selection pressure during human evolution are implicated in diseases such as epithelial cancers, schizophrenia, autoimmune diseases and Alzheimer's disease, all of which differ in prevalence and symptomatology between humans and their mammalian relatives.

In agreement with previous studies, the chimpanzee lineage was found to have more genes under positive selection than any of the other lineages. In addition, we found new evidence to support the hypothesis that genes that have undergone positive selection tend to interact with each other. This is the first such evidence to be detected widely among mammalian genes and may be important in identifying molecular pathways causative of species differences.

**Conclusion:**

Our dataset of genes predicted to have been subject to positive selection in five species serves as an informative resource that can be consulted prior to selecting appropriate animal models during drug target validation. We conclude that studying the evolution of functional and biomedical disease differences between species is an important way to gain insight into their molecular causes and may provide a method to predict when animal models do not mirror human biology.

## Background

Much scientific and medical progress has depended on experimental findings in model organisms being extrapolated to humans. However, even closely related species such as humans and chimpanzees, often experience the same medical condition with varying symptomatology, as seen in cases of Alzheimer's disease or AIDS, or with varying prevalence, for example, autoimmune diseases, epithelial cancers and schizophrenia [1,2].

Comparison of disease prevalence and symptomatology across species is complicated by the fact that modern human lifestyles, very far from the conditions of early human evolution, may reveal susceptibilities to disease that were not evident in the early history of the human species [3]. However, there are observed biomedical differences between humans and other animals that cannot be wholly explained by lifestyle [1,2].

Genetic disease can occur as a by-product of an adaptation which confers a large selective advantage [4]. For instance, the seemingly human-specific disease of schizophrenia [5] and the greater human susceptibility to Alzheimer's disease compared with primates [6] may be a by-product of the human specialisation for higher cognitive function [7]. Besides Alzheimer's disease and schizophrenia, many other diseases also differ in frequency and symptomatology between humans and other mammals. Olsen and Varki [1] and Varki and Altheide [2] list some of these diseases with the emphasis on non-human primates, indicating that for these diseases chimpanzees are not good models despite their close evolutionary relationship with humans. Genes that have been subject to adaptive evolution since the divergence of humans and other primates may be involved in this variation of phenotype and be key to understanding the disease state. Thus, comparative evolutionary genomics can offer insights into these disease mechanisms by correlating molecular differences that arose during species evolution with phenotypic differences in diseases between species; hence elucidating disease-causative genes and pathways.

Direct comparisons of human genomic and transcriptomic information to that of other species reveals three major types of molecular genetic changes which have contributed to species differences. The most obvious mode is the presence or absence of genes in different species, including gene duplication and gene inactivation. Much attention has been paid to genes that are unique to humans or lost in the human lineage [1,2,8,9]. However these probably represent the 'tip of the iceberg' of human genomic differences compared to other species. The second class of molecular genetic changes constitutes of nucleotide substitutions that may cause functional changes in both protein coding and non-coding RNAs. The third category of molecular changes consists of variation in the levels of gene expression between species and in the mechanisms regulating gene expression [8,10].

In this study we investigate the second type of molecular differences, and focus on coding changes in protein-coding orthologous genes. An estimated 70% to 80% of orthologous protein sequences are distinct between humans and chimpanzees [8,9,11]. However, a substantial proportion of differences may have no functional impact on human-specific diseases. Positive selection analyses can determine which nucleotide changes contribute to biological differences between species. This follows from the premise that the action of positive selection pressure in orthologous genes during evolution is often associated with sub- or neofunctionalisation of genes [12]. Determining such genes on the human lineage is thus a rational and promising way to reveal the molecular changes implicated in human-specific disease.

In contrast to previous studies [13-17] which focused on human evolution, the objective of this study was to determine genes which have undergone adaptive evolution in both humans and animal models. We have analyzed alignments of 3079 orthologous genes from human, chimpanzee, mouse, rat and dog to detect signals of positive selection. These species were chosen as they are common models of human disease in medical research and high-quality genomic sequences were available.

Our initial dataset was aggressively filtered to eliminate paralogous alignments, spurious annotations, pseudogenes in one or more species, and poor exon prediction. Hence only quintets for which we could assign orthology with high confidence were used in our analysis for positive selection. Due to this strict screening it must be noted that our orthologue dataset may contain a bias towards orthologues of high levels of conservation, thereby underestimating the number of positively selected genes and underestimating the average levels of divergence. The direction and strength of selection is measured by *ω*, the nonsynonymous to synonymous substitution rate ratio (*d*_N_/*d*_S _= *ω*), with *ω *<1, = 1, and > 1 indicating purifying selection, neutral evolution, and positive selection, respectively. The branch-site model, which tests for positive selection that affects a small number of sites along pre-specified lineages [18-20] was used to test all extant and ancestral lineages for evidence of positive selection. The branch-site model has been shown to be more powerful and more conservative than methods that test positive selection on a given lineage or on a subset of sites [19]. We identified genes predicted to have changed function during mammalian evolution and relate our findings to the diseases known to show biomedical differences between humans and model organisms. These genes may be causative of the phenotypic disease differences between species and are promising targets for therapeutic intervention. This approach is of interest to drug development as detection of positive selection in a drug target or members of a disease pathway may cause animal models to be non-predictive of human biology and explain some observed biomedical differences between species [21].

We found the chimpanzee lineage had many more genes under positive selection than any of the other lineages and three times more than the number of genes in the human lineage. We present evidence to argue against the possibility that this result is due to artefacts introduced by genome sequence coverage, gene sample selection or algorithmic sensitivity to errors in sequence data or alignments. Instead, we conclude that the elevated number of chimpanzee positively selected genes is a true reflection of evolutionary history and is most likely due to positive selection being more effective in the large population sizes chimpanzees have had in the past or possibly remarkable adaptation in the chimpanzee lineage.

As demonstrated in the yeast protein interaction network, evolutionary rate is thought to be correlated with protein connectivity [22-24]. Hence, genes under positive selection are generally believed to be less promiscuous, that is, they interact with fewer genes compared to genes under neutral evolution or negative selection. This may be because promiscuous genes are subject to functional constraints due to their pivotal or multiple roles in biological pathways. However, others analyzing the same data claim that the results are inconclusive [25,26]. We investigate whether genes under adaptive evolution interact with fewer genes compared to genes not under positive selection but did not see a significant difference. However, we also investigated the hypothesis that a gene under adaptive evolution would drive complementary divergence of genes encoding interacting proteins. The most common examples of this co-evolution of interacting genes are receptor-ligand couples that co-evolve to maintain or improve binding affinity and/or specificity. Examples of such genes include the prolactin (*PRL*) gene and its receptor (prolactin receptor, *PRLR*) in mammals [27], primate killer cell immunoglobulin-like receptors (KIRs) that co-evolved with MHC class I molecules [28] and red and green visual pigment genes [29]. Here we present evidence that positively selected genes are significantly more likely to interact with other positively selected genes than genes evolving under neutral evolution or purifying selection.

## Results

### Detection of genes under positive selection

Following multiple hypothesis testing correction (see Methods), a total of 511 Positively Selected Genes (PSGs) were detected. All lineages tested showed significant (*p *< 0.05) evidence of genes evolving under positive selection varying from 54 genes along the human lineage to 162 along the chimpanzee lineage (Table 1). A complete list of PSGs that were detected in each lineage is available in Additional File 1.

**Table 1:** Number of genes under for positive selection in the seven lineages and number of positive genes in OMIM

Lineage	*n*	*m*	*p * value
**Human**	54	8	0.5919
**Chimpanzee**	162	26	0.4190
**Hominid**	56	13	0.0753
**Mouse**	65	11	0.4032
**Rat**	89	18	0.1242
**Murid**	81	21	0.0087*
**Dog**	97	21	0.0577
**All**	511	99	0.0067*

Number of genes under positive selection at *p *< 0.05 (*n*); Number of genes under positive selection in OMIM (*m*) and *p *value from a binomial test to look for over-representation of PSGs within OMIM.

* *p *< 0.01

**Figure 1:**
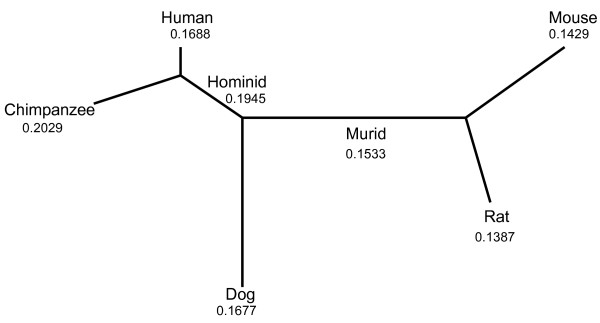
**Five species tree with branch-specific *ω *ratios**. The median *ω *value from free-ratio model estimates of evolutionary rates in 3079 genes for humans, chimpanzees, mouse, rat and dog.

To obtain an overall perspective of the evolutionary rates of the genes in our dataset, the free-ratio model in the codeml program was run on each alignment (see Methods). The median *ω *values for each lineage range from 0.14 in mouse and rat, to 0.17 in human and 0.20 in chimpanzee (Figure 1). Our values for human are comparable to the *ω *values published by the Chimpanzee Sequencing and Analysis Consortium [9] (mouse 0.142, rat 0.137, human 0.208, chimp 0.194) but are more similar to those from Rhesus Macaque Genome Sequencing and Analysis Consortium [30] (human 0.169, chimpanzee 0.175, mouse 0.104), which suggests the strict criteria used to select our input gene set has not introduced a bias for genes with high *ω *values in humans and chimpanzees. The higher median values observed in human and chimpanzee suggest a reduction in purifying selection in hominids.

There were several genes that showed signatures of selection in multiple lineages. We found 17 PSGs along both human and chimpanzee lineages, 8 PSGs along both mouse and rat lineages and 8 PSGs along the hominid and murid lineages. These numbers are significantly greater than we would expect by chance (e.g. there were more genes positively selected in both the human and chimpanzee lineage than would be expected by chance; *p *< 6.864e^-10^, Fisher's test; see Additional File 2, Table 1). Detailed analyses of the genes that overlap between lineages can be found in Additional File 2, 'Genes under selection in adjacent lineages' and Additional File 3.

### Elevated numbers of positively selected genes were detected on the chimpanzee lineage

We found 162 PSGs along the chimpanzee lineage which was three times more than the 54 PSGs detected on the human lineage. This finding was in agreement with other reports of high number of genes that underwent positive selection during chimpanzee evolution [16,31]. Bakewell *et al*. [16] (using a wholly different methodology to this study) identified 21 positive chimpanzee genes and 2 positive human genes from an initial data set of 13,888 genes. Elevated numbers of PSGs along the chimpanzee lineage were also found by Arbiza *et al*. [31] a more similar approach who identified 1.12% of genes under positive selection in the human genome and 5.96% in the chimpanzee genome, which is in close accordance with 1.75% (human) and 5.26% (chimpanzee) obtained here.

### Functional processes affected by positive selection

**Figure 2:**
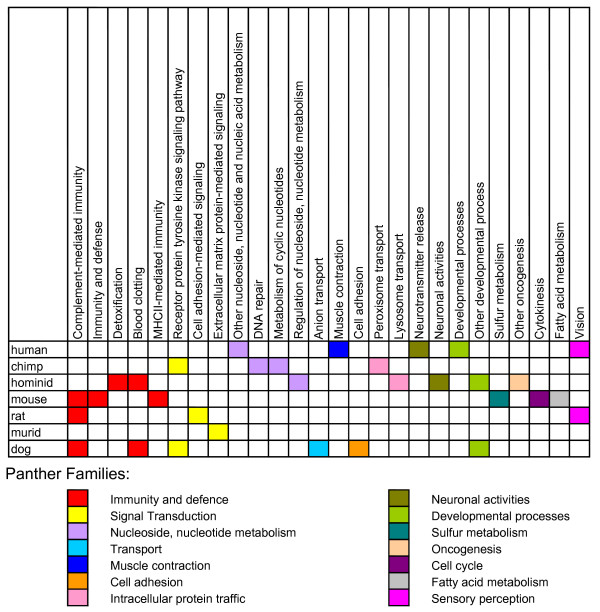
**Biological Process ontologies over-represented by PSGs**. Biological Process ontology terms which had an over-representation of PSGs (*p *< 0.05). Ontology terms are grouped by functional protein PANTHER Biological Process families.

A one-sided binomial test was used to test if the PSGs from each lineage were over-represented among the Biological Process (BP) class of the PANTHER ontology database [32]. The terms that showed the most enrichment were then grouped into BP families (Figure 2) as defined by the PANTHER classification system [33]. Thirty-two BP ontology terms which belong to fourteen BP families were enriched for PSGs (*p *< 0.05, binomial test). After multiple correction, four BP terms were significant at *p *< 0.05. The ontologies that had the most representation by PSGs from the primate lineages were nucleic acid metabolism, neuronal activities, and immunity and defence. Primate PSGs also showed enrichment in functional categories such as development processes or signal transduction, which can be associated with species differences. PSGs from the murid lineages showed over-representation mostly in the functional categories immunity and defence and signal transduction. A significantly high proportion of the chimpanzee PSGs had undefined or unknown biological function (see Additional File 2 'Functional Classification of Chimpanzee PSGs').

### OMIM is enriched for positively selected genes

In order to determine if our dataset of PSGs was significantly enhanced for disease genes, we examined genes that were associated with human diseases as defined by the OMIM, Online Mendelian Inheritance in Man database [34]. Of the 3079 genes used in our analysis, 469 genes (15.2 %) were associated with a disease term in OMIM. Of the 511 PSGs from all seven lineages, 99 genes (19.4 %) were associated with a disease term in OMIM (Table 1). A test based on the binomial distribution showed that there is a significant link between PSGs and disease (*p *= 0.0067). While PSGs along the murid lineage were significantly over-represented in OMIM (*p *= 0.0087), PSGs along the human, chimp or hominid lineages did not display any over-representation (significance cut-off *p *= 0.05).

### No correlation of PSGs and recent selection in human populations

We did not see any evidence of a relationship between a gene being positively selected within human populations and in our mammalian species. In fact, there seems to be a trend that suggests that genes are less likely to have been subject to positive selection along the hominid branch if they were under selection in recent human history. The number of human PSGs was compared with genes shown to be under positive selection pressure within human populations [35]. This is evident in the lower proportion of genes that were both under recent positive selection and positively selected along the human branch (0.03%) compared to the proportion of genes under positive selection along the hominid branch alone (1.8%).

### PSGs on all lineages show evidence of co-evolution

To test if PSGs or proteins encoded by PSGs interact with fewer genes or proteins compared to genes that are not under positive selection, we queried a meta-database of biological interactions (see Methods, [36]) with the list of all PSGs. For the 511 PSGs along all lineages, 155 (30%) did not have any annotated interactions with any other proteins and the median number of interactions was 5. For the 2568 genes in the test set with no evidence of positive selection, 783 (31%) did not have any interactors and the median number of interactors was also 5. Therefore PSGs do not have a lower median number of interactors than genes not under positive selection in the test set (*p *= 0.815; two-tailed Wilcoxon rank sum test), which suggests that number of interactors is not a determinant for PSGs.

To determine if any of the PSGs interact with each other and form smaller clusters of adaptive sub-networks, we queried the same database with the lists of PSGs from each lineage. PSGs from all lineages except the human lineage formed clusters. For example, among the 162 chimpanzee PSGs, 9 clusters were found, consisting of 2 clusters of 3 genes and 7 clusters of 2 genes. We applied a permutation test to determine whether the number and size of the clusters formed is more than would be expected by chance. For PSGs in both the chimpanzee and hominid lineages, the size of the smallest two clusters (chimpanzee clusters 8 (*PEX12, PEX19*) and 9 (*NRP1, MSI1*) and hominid clusters 3 (*DRD2, TH*) and 4 (*ITGAV, AZGP1*)) exceeded what would be expected by chance (*p *< 0.05) (Table 2) and in the dog lineage the third cluster (containing genes *SNTA1, DAG1 *and *MUSK*) was significant, therefore there is some evidence that PSGs are likely to interact and form adaptive sub networks.

**Table 2:** Interacting clusters formed between PSGs on each lineage

Cluster number	Genes in cluster	*p *value for cluster size given previous clusters	*p *value for cluster given number of interactions per gene **
Chimpanzee			
1	*PCSK5, BMP4, PHOX2A*	0.981	0.0013
2	*LHB, OTX1, JUB*	0.391	0.0001
3	*XPC, RAD23A*	0.519	0.0035
4	*NUCB1, PTGS1*	0.346	0.0046
5	*ITGB6, ALOX12*	0.227	0.0030
6	*MYO18A, TRADD*	0.131	0.0028
7	*GSTP1, MAP2K4*	0.075	0.0442
8	*PEX12, PEX19*	0.036*	0.0003
9	*NRP1, MSI1*	0.019*	0.0008
Dog			
1	*CFP, TAL1, SERPINB1, MMP12, PRF1, BCL2, HRG, ITGA5, COMP*	0.385	< 0.0001
2	*CD79A, HCLS1, LCP2*	0.209	0.0012
3	*SNTA1, DAG1, MUSK*	0.036*	0.0002
4	*LRP5, SLC2A2*	0.171	0.0026
5	*ALB, MCAM*	0.082	0.0123
Hominid			
1	*CCL19, CD86, MADCAM1*	0.335	0.0015
2	*MRC2, COL4A4*	0.186	0.0028
3	*DRD2, TH*	0.045*	0.0488
4	*ITGAV, AZGP1*	0.008*	0.0080
Mouse			
1	*HLA-DRB1, HLA-DQA2*	0.755	0.0123
2	*C1R, C1QA*	0.288	0.0030
Murid			
1	*TLR5, CD86, PTGIR*	0.678	0.0001
2	*SCNN1G, SPTA1, HECW1*	0.432	0.0021
3	*CNR1, RAPGEF1*	0.190	0.0110
4	*F5, GP1BA*	0.064	0.0032
Rat			
1	*CDKN2D, TRIM21, CDKN1B, CAST, ICAM1, CFD, ITGB2, C3*	0.360	< 0.0001
2	*KCNA4, ACTN2, PIK3R5*	0.526	0.0016
3	*PIM1, RP9*	0.280	0.0063
4	*ASPH, HDAC4*	0.118	0.0053

*p < 0.05.

** All tests to investigate if the size of the cluster would be more than expected by chance given the number of interactors each individual gene in that cluster were significant (*p *< 0.05).

We also tested each cluster to determine whether the size of the cluster is more than would be expected by chance given the number of interactors for each individual gene in the cluster. All 28 clusters were found to be significant (*p *< 0.05 by permutation test) (Table 2), therefore there is a significant phenomenon of PSGs interacting with other PSGs. To confirm this observation, a further analysis was performed on the genes that interact with the beta 2 integrin gene (*ITGB2*) which showed evidence of positive selection along the rat (*p *< 0.001) and murid (*p *< 0.05) lineages. Three of its four known interacting alpha subunits [37] also showed positive selection either on the murid branch (*ITGAL*, *p *< 0.01; *ITGAX*, *p *< 0.05) or on the mouse branch (*ITGAD*, *p *< 0.001).

## Discussion

The functional categories enriched for PSGs in this study were found to closely correlate with those detected in previous genome scans [38]. The consensus is compelling given the different techniques used in each study and the risk of false positives inherent in large-scale studies. It is interesting to note that among the five species analyzed, protein families with distinct functions could be identified as evolving under positive selection for each species. Molecular changes in these genes are potentially responsible for driving the species-specific differences.

### Hypotheses to explain the high number of PSGs on the chimpanzee lineage

The high number of PSGs along the chimpanzee lineage cannot be explained by the incorrect calling of orthologues or alignment quality, as we employed conservative filters during the orthologue calling procedure and manually checked all the PSG alignments. We also checked the underlying genomic quality values for the chimpanzee PSGs and only 1 sequence had quality values less than Q20 (error rate of 0.01) among the sites predicted to be under positive selection and hence the high number of PSGs is not due to poor genomic sequence quality. However, we acknowledge that the chimpanzee genome sequence is unfinished and will contain errors and rare polymorphisms, as exemplified by its occasional mismatches to mRNA and gene prediction sequences (such as those provided by RefSeq). In this study, we have tried to minimise the effect of sequence error by preferentially using validated gene sequences when available and high quality genome sequence when not. Nevertheless, we cannot exclude sequence error as a factor in our results. Therefore, we also checked that taxon sampling did not affect the number of PSGs on other lineages and hence ensured that quality issues from one species did not affect the signals for positive selection on other lineages (see Additional File 2 'Taxon sampling does not affect detection of positive selection' and Additional File 4). Additionally, comparison of 11 of the extremely divergent chimpanzee sequences to their orthologues in other primates (marmoset, macaque and orang-utan) (see Additional File 2 'Chimpanzee PSGs are lineage-specific') showed that the amino acid differences observed in the 11 chimpanzee sequences are specific to the chimpanzee, with the other primate sequences having the same state as the human sequence.

One likely explanation for the high number of PSGs in the chimpanzee lineage could be the reported high polymorphism in the individual chimpanzee sequenced (heterozygosity rate of 9.5 × 10^-4 ^[9]). This rate is slightly higher than what was seen among West African chimpanzees (8.0 × 10^-4 ^[9]) which have similar diversity levels to that seen in human populations [39]. Population size is another possible explanation as positive selection may have had a reduced efficacy in humans than in chimpanzees due to the larger long-term population size of chimpanzees compared to humans indicated by reduced nucleotide diversity and elevated polymorphism among chimpanzee sequences [40].

### PSGs implicated in diseases with biomedical differences between mammals

Overall, we observed that PSGs were over-represented among genes found in OMIM. Yet in contrast to the findings of Clark *et al*. [14], PSGs along the human lineage were not seen to display any over-representation in OMIM. Our findings, however, were consistent with other recent studies that found no significant associations [9] or only marginal associations [16] between human PSGs and human diseases. The OMIM database is the most complete freely available source of disease associated genes available but does include genes associated with non-pathological conditions such as hair colour; hence noise from such data might lead to non-significant results during statistical tests. Tests for enrichment of PSGs within more precise collections of disease genes may yield different results.

Examination of individual PSGs along the human and hominid lineages, revealed genes implicated in diseases that show biomedical differences between mammals. Below we illustrate how some of the human and hominid PSGs identified in our study are linked to medical conditions described as being more prevalent or having increasing severity in humans compared to apes [1,2].

### Epithelial cancers

Human epithelial cancers are thought to be the cause of over 20% of deaths in modern human populations whereas among non-human primates, the rates are as low as 2–4% [41]. Although this may be partly attributed to carcinogenic factors in the lifestyles of modern humans and differences in life expectancy, there are many intriguing lines of evidence to suggest that another overwhelming factor is the presence of susceptibility genes in human [8,42-47].

Among the human lineage PSGs detected here a number of genes have been implicated in the development of epithelial cancers:

• ***MC1R ***(melanocortin-1 receptor) modulates the quantity and type of melanin synthesised in melanocytes. Mutations in this gene have been associated with melanomas [48]. An allele of this gene associated with pale skin colour and red hair, was recently located in the Neanderthal sequence [49] which suggests that this gene was also under recent selection in human evolution. Functional changes in the human *MC1R *gene which causes a change in skin colour could lead to an increased susceptibility to ultra-violet radiation and hence higher levels of melanoma in humans.

• The G-protein coupled receptor ***EDNRB ***(endothelin type-B receptor) and its physiological ligand, endothelin 3, are thought to play key roles in the development of melanocytes and other neural crest lineages [50]. *EDNRB *promotes early expansion and migration of melanocyte precursors and delays their differentiation. *EDNRB *is greatly enhanced during the transformation of normal melanocytes to melanoma cells where it is thought to play a role in the associated loss of differentiation seen in melanoma cells [51].

• The presence of the ***ALPPL2 ***gene product, an alkaline phosphatase isoenzyme, has been shown to increase the potential of premeiotic male germ cells to malignant transformation. Increased promoter activity of this gene was seen in the process of tumour progression. *ALPPL2 *has now been confirmed as a marker for testicular germ cell tumours [52].

• ***GIPC2 ***mRNAs are expressed in cells derived from a diffuse-type of gastric cancer, and also shows increased expression in several cases of primary gastric cancer [53]. The PDZ domain of the *GIPC2 *protein interacts with several genes that are involved in modulation of growth factor signalling and cell adhesion (e.g. *FZD3*, *IGF-1 *and NTRK1). Thus *GIPC2 *may play key roles in carcinogenesis and embryogenesis.

In the hominid lineage, several PSGs have also been implicated in epithelial cancer development suggesting differences in cancer disease processes between hominids and other mammals:

• ***MSH2 ***is a DNA mismatch-repair gene that was identified as a common locus in which germline mutations cause hereditary nonpolyposis colon cancer (HNPCC) [54]. As deficiencies in any DNA repair gene would potentially increase cancer risk, this group of genes is of interest in investigation of species differences in cancer prevalence. We found that genes which are involved in DNA repair and nucleotide metabolism were over-represented for PSGs along the chimpanzee and human lineages respectively (Figure 2). Enrichment of PSGs within the nucleotide metabolism category has also been reported previously [38].

• The*** ABCC11 ***[ABC-binding cassette, subfamily C, member 11] gene product is highly expressed in breast cancer compared to normal tissue. *ABCC11 *is regulated by ERα, which mediates the tumour promoting effects of estrogens in breast cancer [55].

### Ataxia and Migraine

The calcium channel gene, ***CACNA1A***, was found to be under positive selection along the human lineage. In humans, mutations in *CACNA1A *are associated with channelopathies, such as spinocerebellar ataxia 6 and episodic ataxia type 2 [56] as well as with more prevalent conditions such as familial hemiplegic migraine, dystonia, epilepsy, myasthenia and even intermittent coma [57]. It is possible that the trafficking or signal modulation of *CACNA1A *differs between humans and other mammals as a result of adaptation of the central nervous system, which could result in humans being more prone to these neurological disorders. The benefits of enhanced CNS excitability may outweigh the risk of severe headache and disability, the symptoms of migraines [58]. It could also be an artefact of design constraints in the brain resulting from imperfect interconnections between older and more recently evolved brain structures [4].

### Alzheimer's disease

A gene implicated in Alzheimer's disease [59,60], ***APOE***, was under positive selection along the hominid lineage. Selection for functional changes of the *APOE *gene in the hominid lineage could be related to either its role in neurological development or in lipid metabolism. Of the eight amino acids found to be under positive selection in this study, four are present in the lipid-binding carboxyl terminus.

The suggestion that there are species differences in Alzheimer's disease between humans and other mammalian species comes from the lack of pathological lesions including the neurofibrillary tangles associated with human Alzheimer's disease being observed in the brains of elderly chimpanzees [6,61] or elephants [62]. Also, transgenic mouse models of Alzheimer's disease that presented β-amyloid neuropathology do not exhibit the cognitive decline at the first appearance of amyloid plaques seen in humans [63]. Finally and intriguingly, mammals other than humans seem to have just one allelic form of *APOE*, the E4 allele [60,64], the same form in humans predisposes carriers to a much higher risk of Alzheimer's disease [65].

We hypothesise that the positive selection pressure acting on *APOE *during hominid evolution changed the role of *APOE *in neurological development, presumably in concert with the expansion of cognitive ability. However, alternative studies have suggested that the major evolutionary events associated with cognition have occurred much earlier [66]. A consequence of increased cognitive ability maybe increased susceptibility to dementing diseases such as Alzheimer's disease [67] but as the onset of these diseases is past reproductive age, these diseases would be overlooked by natural selection. The other possibility is that dietary pressures influenced the evolution of *APOE *in mammals, with species adapting to diets with differential levels of lipids and so favouring different forms of APOE [68].

### Schizophrenia

Neurological studies have shown that brain areas differentially dysregulated in schizophrenia are also subject to the most evolutionary change in the human lineage [69]. A number of PSGs along the human lineage are associated with schizophrenia:

• SNPs in the gene ***PIK3C2G ***[phosphoinositide-3-kinase] have been shown to be associated with schizophrenia recently [70]. This gene is related to the phosphoinositide pathway, and thus is a probable candidate for schizophrenia and bipolar disorder [71].

• Another candidate for chronic schizophrenia is the Q399 allele of the ***XRCC1 ***protein, which plays a role in base excision repair [72]. The pathophysiology of schizophrenia is associated with an increased susceptibility to apoptosis. Mutations in *XRCC1 *may cause DNA damage which if detected cause apoptosis regulators to arrest cell cycle progression.

### Other cognitive disorders

Also subject to positive selection along the human lineage was the gene ***GFRA3***, a receptor for artemin and a member of the glial cell line-derived neurotrophic factor (GDNF) family of ligands. This gene acts as a signalling factor regulating the development and maintenance of many sympathetic neuronal populations [73]. In particular, along with other GDNF family members, artemin plays a role in synaptic plasticity, a mechanism thought to be central to memory [74]. Deficiencies in *GFRA3 *would be expected to cause cognitive impairment making it a candidate gene for cognitive disorders.

### Autoimmune diseases

Autoimmune diseases are rare in non-human primates whereas they are relatively common in humans [41]. ***CENP-B ***is one of three centromere DNA binding proteins that are present in centromere heterochromatin throughout the cell cycle. Autoantibodies to these proteins are often seen in patients with autoimmune diseases, such as limited systemic sclerosis, systemic lupus erythematosus, and rheumatoid arthritis [75]. The positive selection pressure acting on this gene during human evolution is consistent with experimental results that antigenic specificity in the C-terminus of *CENP-B *is species-specific [76].

### Positive selection of regulatory genes

Selection events on coding sequences may also have effects on gene expression regulation. One transcription factor that showed signs of positive selection along the human lineage was ***HIVEP3 ***(immunodeficiency virus type I enhancer binding protein 3). This gene belongs to a family of zinc-finger proteins whose functions include activating HIV gene expression by binding to the NF-kappaB motif of the HIV-1 long terminal repeat [77]. It is commonly known that HIV infection in chimpanzees does not progress to the level of medical complexity that is seen in human AIDS [41]. In chimpanzees the virus lives in a benign relationship within the immune system whereas in humans it infects and destroys helper T-cells. Functional changes in transcription factors such as *HIVEP3 *between humans and chimpanzees could explain the observed differences in HIV disease progression.

Regulatory elements of gene expression also showed evidence of positive selection along the human lineage. One is the ***MOV10 ***gene (Moloney leukaemia virus 10, homolog), an RNA helicase contained in a multiprotein complex along with proteins of the 60S ribosome subunit. *MOV10 *is associated with human RISC (RNA-induced silencing complex) [78]. RNA silencing or interference (RNAi) has been recently described as an important therapeutic application for modulating gene expression at the transcript level or for silencing disease-causing genes [79,80]. Any functional changes in the *MOV10 *gene due to selection may affect transcriptional control of multiple genes and would therefore prompt widespread differences among species.

## Conclusion

We conclude that comparative evolutionary genomics has an important contribution to make to the study of mammalian disease, enabling identification of candidate genes for further *in vivo *investigation. Researchers traditionally see the biomedical differences between humans and model organisms as an obstacle to progress. However, we propose these differences also provide an opportunity to dissect the molecular causes of disease. To take advantage of this opportunity, we need powerful computational evolutionary algorithms (such as used in this study) and a robust approach to utilise the ever-expanding genomic sequence data. Two major challenges inherent to this approach are: firstly, sequence errors are likely to increase the false positive rates in identifying cases of positive selection pressure and secondly, to fully utilize this information requires detailed accounts of the physiological differences in disease occurrence and symptomatology between species which are currently sparse.

Understanding the evolutionary history of disease genes can also significantly impact the choice of pre-clinical animal models in the drug discovery process [81]. The success rates in pharmaceutical pipelines remains low, one reason being the difficulty in successfully translating safety and efficacy studies from animal models to humans. Pre-clinical studies assume that drug targets in the experimental species and in humans are functionally equivalent, which is not always the case [38]. In particular, animal models of neurodegenerative diseases have been shown to lack predictive validity in humans [82]. Studies of selection pressure during gene evolution can provide valuable information for the choice of animal models for drug target validation. Our results of PSGs in the five mammalian species serve as an informative resource that can be consulted prior to selecting appropriate animal models during drug target validation in the pharmaceutical industry.

Positive selection pressure would be expected to act not just on one gene at a time but on pathways of genes. We found that genes that were subject to positive selection along the same lineage were significantly more likely to interact with each other than with genes not under positive selection, the first evidence for co-evolution of genes as a widespread phenomenon in mammals. We suggest that the high level of connectivity between PSGs is caused by compensatory change of a protein's interaction partners when a protein undergoes change in response to selection.

We observe many chimpanzee genes which have been subject to positive selection during the evolution of their anthropoid ancestor. Since medical research and the vast majority of biological research have been focussed on discovering more about human biology, we know a lot less about chimpanzee-specific characteristics. The number of PSGs on the chimpanzee lineage would suggest that these chimpanzee adaptations are at least as striking as our much-vaunted human-specificities.

## Methods

### Sequence data

We analysed all Entrez human genes (accessed in September 2006) that were annotated as protein coding and had a confirmed mRNA sequence. The longest open reading frame associated with each gene was included in the starting set. Curated mRNA sequences from the RefSeq NCBI database and genomic sequences for the four model organisms (chimpanzee, mouse, rat and dog) and chicken (outgroup) were extracted from GenBank (accessed in September 2006).

### Orthologue calls

The orthologue detection pipeline used reciprocal tBlastX searches [83] between the human and model organism sequence databases. If the highest scoring non-human species sequence was genomic, indicating an mRNA sequence was not available for this gene in this species, it was processed via GeneWise [84] to identify a predicted gene structure and remove introns, using the human peptide as template. The resulting cDNA sequence was then used as a query in the reciprocal tBlastX search against the human database. Highest scoring mRNA sequences were submitted to the reciprocal tBlastX search without modification.

Reciprocal best hits between the human gene and the model organism gene were marked as the orthologue pair for that human transcript query on the condition that the log of the *p *value from the best hit of the human mRNA sequence against the model organism database was higher than 95% of the log of the *p *value of the best hit from the reciprocal step.

Incomplete genome sequencing will also contribute to error in orthologue calling. Reciprocal blasting is invalidated as a method for calling orthologues in these circumstances as the absence of the true orthologue would cause a more divergent paralogue to be the top hit. To address this problem we added a cut-off, which required the *p *value of the putative orthologue for that species to be less than that of the chicken orthologue for that gene. The chicken was chosen because it was the closest relative to mammals for which a complete draft genome sequence was available at sufficient coverage [85]. For the 262 human genes with no chicken orthologue, those predicted by reciprocal BLAST alone were analysed but these genes were flagged as potential problems.

### Detecting genes affected by positive selection

The resulting sets of 5 orthologous sequences were translated and aligned using Muscle [86], then converted to corresponding nucleotide alignments. All alignments were then corrected for frameshifts in the sequences from the model organisms relative to human. Unrooted tree files for each alignment were created using a standard mammalian species tree [87] ((human, chimpanzee), (mouse, rat), dog) (Figure 1). Initially, data sets were analyzed using the M0 (one-ratio) model implemented in the codeml program from the PAML package [88]. The M0 model assumes constant *ω *ratio for all branches in the tree and among all codon sites in the gene [89]. Two runs of the M0 model were performed on each alignment to check that values for log-likelihood, *κ *and branch lengths were consistent between the two runs. Runs that were not consistent were rerun until the values converged. In the subsequent analyses using the branch-site model, the branch lengths and the transition/transversion rate ratio *κ *were fixed to their estimates under the M0 model. This strategy reduces the computation time as the number of parameters to be estimated is reduced.

To infer the lineage specific evolution of genes, the branch-site model [18,19] was used to test for positive selection. We tested each of the seven branches on the species phylogeny, treating each in turn as the foreground branch. Results prior to multiple hypothesis correction should not be used for subsequent analysis as the family-wise error rate is unacceptably high [90]. Here we report results following a Bonferroni correction for multiple testing which is known to be conservative and hence, prediction of positive selection is particularly robust. The corollary of such a strict approach is the potential generation of false negatives. The alternative branch-site model has four codon site categories, the first two for sites evolving under purifying selection and neutral selection on all the lineages and the additional two for sites under positive selection on the foreground branch. The null model restricts sites on the foreground lineage to be undergoing neutral evolution. Each branch-site model was run at least three times to ensure convergence of log-likelihood values at or within 0.001. Runs that did not converge with additional runs indicated problems with the data and reported as such.

### Data Curation

When the data from the automated procedures was examined closely, it was noted that some alignments had areas of ambiguous alignment or areas where sequences did not appear orthologous. Areas of non-orthology could result from incomplete gene predictions due to gaps in the genomic sequence or absent or variant exons. Therefore the data were subjected to further manual corrections detailed below:

1. To correct for regions of low similarity, all alignments were scanned to mask out parts of a sequence where > 3 consecutive codons were different to the other sequences in the alignment and where these codons were flanked by gaps on one or both sides. Sequences that also contained frameshifts relative to the human sequence were corrected.

2. After re-running PAML on the entire dataset, we manually examined the alignments of all significant results (*p *< 0.05). The result was discarded if the gene sequence belonging to the lineage that was identified as being under positive selection had a frameshift or was ambiguously aligned.

### Analysis of interaction data

A network consisting of protein-protein interactions such as binding and phosphorylation, transcriptional control and post-translational modification was used to search if genes under positive selection interact together. Interaction data in the network was licensed from several commercial vendors including Ingenuity [91], Jubilant [92], GeneGO [93], NetPro [94] and HPRD [95]. All of the information from these databases is based on manual curation of literature. In addition, high-quality, automatically extracted interactions licensed from the PRIME database [96] were also included in the network. Interactions associated with transcriptional regulation were obtained from experimental validation protein-DNA binding relationships licensed from the TransFac [97] and TRRD [98] databases. No distinction is made between DNA, RNA and protein for a particular gene, and all three are represented as a single node in the network. Searches of gene lists that resulted in a biological sub-network were conducted and scored as in [36].

## Abbreviations

PSG: Positively Selected Gene.

## Authors' contributions

JJV participated in the phylogenetic analysis, data analysis and QC, and helped draft the manuscript. SH wrote scripts for data QC and helped draft the manuscript. RDE conceived of the study, participated in phylogenetic analysis and helped draft the manuscript. HAM conceived of the study, participated in design of orthologue calling pipeline, data QC strategy and analysis of results and reviewed the manuscript. DR designed and participated in the co-evolution experiments. SDT designed and participated in data collection and orthologue calling pipeline. VK designed the orthologue calling pipeline. MW wrote scripts for data collection. MDS wrote scripts for data collection. SMF helped draft the manuscript. PS helped draft the manuscript. ZY participated in the phylogenetic analysis, data analysis and helped draft the manuscript. JDH conceived of the study, participated in the phylogenetic analysis, data analysis and co-evolution experiments and helped draft the manuscript.

## Supplementary Material

Additional file 1 **Names of genes under positive selection in each lineage.** Entrez gene names of positively selected genes in each of the seven lineages. Click here for file

Additional file 2 **Description of results from additional analyses.** Additional work carried to confirm results in the main text are described and discussed. Click here for file

Additional file 3 **PSGs along the hominid and murid lineages cluster to form networks involved in inflammatory processes.** Network diagrams of positively selected hominid and murid genes that interact together and are involved in inflammatory functions. Click here for file

Additional file 4 **Summary of results from taxon exclusion studies.** Circle representation of genes significant in one or more of the permutation studies. Click here for file
