# Supplementary material for: The role of positive selection in determining the molecular cause of species differences in disease
Source: BMC Evol Biol. 2008 Oct 6;8:273. doi: 10.1186/1471-2148-8-273 (PMC2576240; doi:10.1186/1471-2148-8-273)
Supplement: Additional file 1 — Names of genes under positive selection in each lineage. Entrez gene names of positively selected genes in each of the seven lineages. [file 1471-2148-8-273-S1.doc]

**Additional File 1: Names of genes under positive selection in each lineage.**

| **Human** | **Chimpanzee** | **Hominid** | **Mouse** | **Rat** | **Murid** | **Dog** |
| --- | --- | --- | --- | --- | --- | --- |
| ABCF1 | ABCF1 | ABCC11 | ADMR | ABTB2 | ABCB10 | ALB |
| ALPPL2 | ACTN2 | ADAD2 | AQP9 | ACTN2 | ACRBP | ALS2CL |
| ANGEL1 | ACVRL1 | ADRB2 | AVPR1B | AIM1 | ADRB3 | APBB1 |
| ANKRD35 | ADCY5 | AMAC1 | C11ORF34 | APOF | ARMC3 | B4GALT4 |
| ARID2 | ADCY6 | APOE | C19ORF16 | ARHGAP27 | BLVRA | BCAS1 |
| ATPBD3 | ALG10 | AZGP1 | C1QA | ARHGEF17 | C10ORF88 | BCL2 |
| C8ORF42 | ALOX12 | C11ORF34 | C1R | ASPH | C10ORF93 | BCL3 |
| CA14 | ALPPL2 | C18ORF34 | C20ORF102 | ATP11C | C1ORF156 | BMS1 |
| CACNA1A | ANGEL1 | C1QA | C20ORF186 | ATRX | C5ORF32 | C11ORF34 |
| CACNA1S | ANKRD35 | C9ORF75 | CA6 | C11ORF34 | C6ORF170 | C12ORF34 |
| CEACAM20 | AQP2 | CCL19 | CCDC83 | C19ORF16 | C6ORF194 | C15ORF27 |
| CENPB | ARHGEF17 | CD86 | CCDC95 | C3 | CACNA1A | C20ORF186 |
| CNGA4 | ARMC3 | CDC42EP2 | CD86 | C8B | CCDC73 | C6ORF182 |
| COL11A1 | ARMCX5 | CDKN2B | CDC14B | CA6 | CD86 | C8A |
| CTAGE6 | ARRB1 | CLSTN2 | CENPC1 | CARD11 | CDCA2 | CACNA1S |
| EDNRB | ATP6AP1 | COL11A1 | DHDH | CAST | CLSTN2 | CCDC66 |
| EMB | BLK | COL4A4 | DOCK3 | CCDC108 | CNR1 | CD79A |
| FLJ40722 | BMP4 | COMP | DSPP | CCDC18 | CX62 | CDCA2 |
| GFRA3 | C10ORF93 | CXYorf1 | FAIM3 | CCDC7 | CXCL13 | CDH17 |
| GIPC2 | C11ORF24 | DRD2 | FLJ40722 | CDC14B | DAG1 | CDH22 |
| GPR111 | C14ORF39 | EMP1 | FLT1 | CDH22 | EFCAB5 | CFP |
| GPR83 | C16ORF48 | ENG | FZD6 | CDKN1B | ELOVL4 | CLCN1 |
| GPRC6A | C17ORF28 | ENSA | GIMAP8 | CDKN2D | ENAM | COMP |
| HIVEP3 | C1ORF129 | F5 | GPR83 | CFD | ETV2 | CRB2 |
| IFRD2 | C1ORF174 | FLJ46266 | H6PD | CHRNA7 | F5 | CREBL1 |
| INPP5B | C21ORF13 | FZD2 | HECW1 | CILP | FZD2 | DAG1 |
| KCNK5 | C3 | GDPD4 | HLA-DQA2 | COL11A1 | GAS2L2 | DBX1 |
| KIAA0372 | C8ORF42 | GIPC2 | HLA-DRB1 | CYB561 | GJC1 | DPP6 |
| LOC388969 | CCDC27 | GPR116 | HOXC6 | DAGLB | GP1BA | DSPP |
| LOC389072 | CCDC88C | GPR97 | IZUMO1 | DHDH | GPR1 | EFCAB4B |
| LOC619207 | CCDC97 | GSTO2 | KIAA1949 | DNM1 | GPR111 | ENSA |
| MC1R | CDH15 | HSPA1B | KLF11 | DPP6 | GPR113 | EPHA1 |
| MGC50722 | CHKA | HTR1D | KRT2 | DSC2 | HBD | EVI2A |
| MICALCL | CLTB | HTR2C | LOC253012 | EIF2C3 | HECW1 | F5 |
| MOV10 | CNGA4 | ITGAV | LOC388323 | FLJ13305 | HLA-C | FGF20 |
| MYF5* | COL11A2 | LOC220686 | LOC497190 | FLJ40722 | HOXA11 | FLJ45187 |
| NR5A1 | COMP | LOC619207 | MARCH3 | FXYD1 | HRH2 | GALNS |
| OR4F17 | CPNE9 | MADCAM1 | MGC71993 | FZD2 | HSPA1A | GAS2L2 |
| PDE6A | CSTF1 | MCAM | MMPL1 | GPR141 | HSPE1 | GDPD4 |
| PIK3C2G | CXORF38 | MMPL1 | MRVI1 | HDAC4 | IFIT2 | GGTLA1 |
| RBM16 | DBX1 | MRC2 | MYH15 | HLA-B | INSL3 | GPRASP1 |
| RDM1 | DIP2C | MSH2 | NDUFC1 | ICAM1 | ITGAV | GRID1 |
| REPIN1 | DOPEY1 | MYCT1 | NLRP9 | IMPG1 | KRT2 | GRM3 |
| RKHD1 | DUSP2 | NRAP | NOVA2 | INPP4A | KRTAP3-3 | HADHB |
| RUFY4 | DYRK2 | NUDT22 | NXPH4 | IQSEC3 | LAMC2 | HCLS1 |
| SLC5A9 | EEF1G | PHYHD1 | PHYH | IQUB | LIPC | HDAC4 |
| SRL | EFCAB4A | RUFY4 | PZP | ITGB2 | LYZ | HDC |
| ST8SIA3 | EFCAB4B | SCML4 | RAB11FIP2 | KCNA4 | MAGEB4 | HLA-DMB |
| TMPRSS12 | EHHADH | TFF1 | RAPGEF2 | KIFAP3 | MAST3 | HRG |
| TRIM67 | ELF4 | TFPT | RRAGA | KRT31 | MCOLN2 | HSPA6 |
| UMPS | EMD | TH | SASP | LASS2 | MDC1 | IFT88 |
| XRCC1 | ENTPD5 | TRAF6 | SCD | LCTL | MRC2 | IL18RAP |
| ZNF324B | EOMES | TXNDC3 | SEPT1 | LDHD | MRPL54 | INPP5B |
| ZRSR2 | ETAA1 | WDR42B | SERINC5 | MAN1A2 | NLGN4Y | ITGA5 |
|  | FAM134A | ZNF384 | SH2D6 | MSL-1 | NLRP5 | ITPKA |
|  | FLRT1 | ZNF665 | SLC1A5 | NKX2-5 | NLRP9 | KIAA1727 |
|  | GALNT6 |  | SSTR2 | LOC619207 | NR1I2 | KRTAP2-4 |
|  | GDPD4 |  | STS | OPN5 | NUF2 | LCP2 |
|  | GFPT2 |  | SYCP3 | PCDHB14 | OR7C1 | LRP5 |
|  | GIPC2 |  | SYT4 | PDE6C | OXSM | MCAM |
|  | GIYD1 |  | TARP | PELI3 | PHACTR1 | MDGA1 |
|  | GPC3 |  | TIMD4 | PIK3R5 | PHYH | MGC50722 |
|  | GPD1L |  | TMF1 | PIM1 | PNLIP | MMP12 |
|  | GPR19 |  | TST | PLIN | PSMB6 | MTDH |
|  | GPX2 |  | UNQ9438 | PLXNC1 | PTGIR | MUSK |
|  | GRIK5 |  |  | PRSS1 | RAPGEF1 | NLRP5 |
|  | GSTP1 |  |  | PRSS35 | RBM16 | NOS1AP |
|  | HCRTR1 |  |  | PRSS36 | RP5-1054A22.3 | NRTN |
|  | HLA-DRB1 |  |  | PSMB4 | SAFB | PALM2-AKAP2 |
|  | ICA1L |  |  | RAB11FIP3 | SCNN1G | PAX1 |
|  | IGFALS |  |  | RGSL1 | SLC34A3 | PCDHB6 |
|  | INPP5B |  |  | RP9 | SNRPA | PCTK2 |
|  | IRAK2 |  |  | RRAGA | SPTA1 | PDE6C |
|  | ISG15 |  |  | RS1 | TAS2R39 | PLA1A |
|  | ITGB6 |  |  | SLCO2A1 | TLR5 | PRF1 |
|  | JUB |  |  | STON1 | TRPC3 | PTGFRN |
|  | KIAA0372 |  |  | STS | TXNDC3 | PTX3 |
|  | KRT15 |  |  | SYT4 | VGLL2 | RASGRF2 |
|  | KRT34 |  |  | TAC4 | ZC3H6 | SCN8A |
|  | LGALS7 |  |  | TARP | ZNF658B | SEPP1 |
|  | LHB |  |  | TEKT4 | ZNF665 | SERPINB1 |
|  | LOC553158 |  |  | THEM5 |  | SIDT1 |
|  | MAGEH1 |  |  | TMEM162 |  | SIGLEC5 |
|  | MAP2K4 |  |  | TRIM21 |  | SIRT1 |
|  | MAPK4 |  |  | UBR1 |  | SLC17A8 |
|  | MAST3 |  |  | UNC13A |  | SLC22A18 |
|  | MFAP4 |  |  | ZBTB38 |  | SLC26A2 |
|  | MGC50722 |  |  | ZNF43 |  | SLC2A2 |
|  | MICALCL |  |  | ZNF780B |  | SLC31A1 |
|  | MIPEP |  |  |  |  | SLCO4C1 |
|  | MORC2 |  |  |  |  | SNTA1 |
|  | MSH2 |  |  |  |  | TAL1 |
|  | MSI1 |  |  |  |  | TRY1 |
|  | MYO18A |  |  |  |  | UGCGL1 |
|  | MYO1A |  |  |  |  | XRCC1 |
|  | NLRC3 |  |  |  |  | ZFP36 |
|  | NPR1 |  |  |  |  | ZNF282 |
|  | NTSR1 |  |  |  |  |  |
|  | NUCB1 |  |  |  |  |  |
|  | OR4F17 |  |  |  |  |  |
|  | OTX1 |  |  |  |  |  |
|  | PAK2 |  |  |  |  |  |
|  | PCSK5 |  |  |  |  |  |
|  | PEX12 |  |  |  |  |  |
|  | PEX19 |  |  |  |  |  |
|  | PHOX2A |  |  |  |  |  |
|  | PI16 |  |  |  |  |  |
|  | PIGV |  |  |  |  |  |
|  | PIK3C2G |  |  |  |  |  |
|  | PPP2R1A |  |  |  |  |  |
|  | PSD2 |  |  |  |  |  |
|  | PSMB4 |  |  |  |  |  |
|  | PTGS1 |  |  |  |  |  |
|  | RAD23A |  |  |  |  |  |
|  | RBM16 |  |  |  |  |  |
|  | RNF10 |  |  |  |  |  |
|  | RNF145 |  |  |  |  |  |
|  | RUFY4 |  |  |  |  |  |
|  | SAFB |  |  |  |  |  |
|  | SALL1 |  |  |  |  |  |
|  | SCUBE3 |  |  |  |  |  |
|  | SERINC2 |  |  |  |  |  |
|  | SERINC5 |  |  |  |  |  |
|  | SERPINA5 |  |  |  |  |  |
|  | SH3PXD2B |  |  |  |  |  |
|  | SLC14A1 |  |  |  |  |  |
|  | SLC22A18 |  |  |  |  |  |
|  | SLC45A1 |  |  |  |  |  |
|  | SMC3 |  |  |  |  |  |
|  | SNAPC1 |  |  |  |  |  |
|  | SPATA1 |  |  |  |  |  |
|  | SPATA21 |  |  |  |  |  |
|  | SPERT |  |  |  |  |  |
|  | SPR |  |  |  |  |  |
|  | SREBF2 |  |  |  |  |  |
|  | SYNC1 |  |  |  |  |  |
|  | TBC1D10C |  |  |  |  |  |
|  | TEF |  |  |  |  |  |
|  | TEX264 |  |  |  |  |  |
|  | TFR2 |  |  |  |  |  |
|  | TKTL1 |  |  |  |  |  |
|  | TLE2 |  |  |  |  |  |
|  | TLR5 |  |  |  |  |  |
|  | TMEM175 |  |  |  |  |  |
|  | TPCN2 |  |  |  |  |  |
|  | TRADD |  |  |  |  |  |
|  | TRIM65 |  |  |  |  |  |
|  | UGT1A8 |  |  |  |  |  |
|  | UPK3A |  |  |  |  |  |
|  | USP54 |  |  |  |  |  |
|  | VMO1 |  |  |  |  |  |
|  | WDR27 |  |  |  |  |  |
|  | WDR34 |  |  |  |  |  |
|  | WDR90 |  |  |  |  |  |
|  | XPC |  |  |  |  |  |
|  | ZFP36L1 |  |  |  |  |  |
|  | ZNF289 |  |  |  |  |  |
|  | ZNF324B |  |  |  |  |  |
|  | ZNF43 |  |  |  |  |  |
|  | ZNF653 |  |  |  |  |  |
|  | ZNF768 |  |  |  |  |  |
|  | ZRSR2 |  |  |  |  |  |

*During the review stage of this manuscript the human sequence for MYF5 (NP_005584.2) was revised so that the human and chimpanzee protein sequences became identical, suggesting that the positive selection detected for this gene in the human lineage may be spurious due to data quality.
