# Supplementary material for: The role of positive selection in determining the molecular cause of species differences in disease
Source: BMC Evol Biol. 2008 Oct 6;8:273. doi: 10.1186/1471-2148-8-273 (PMC2576240; doi:10.1186/1471-2148-8-273)
Supplement: Additional file 2 — Description of results from additional analyses. Additional work carried to confirm results in the main text are described and discussed. [file 1471-2148-8-273-S2.doc]

**Additional File 2: Description of results from additional analyses**

**Genes under selection in adjacent lineages**

The genes under selected in adjacent lineages (Table 1) suggest the presence of some common targets of positive selection in each of the pairs of species and may represent malleable genes that are involved in adapting to changing external environments, like sensory perception and dietary content. Some examples of such genes are the olfactory receptor *OR4F17*, a PSG in both the human and chimpanzee lineages, which illustrates what is known about both humans and chimpanzees showing extensive evidence of olfactory adaptation [1]. *DHDH*, a PSG in both the mouse and rat lineages, is involved in carbohydrate metabolism which may be an adaptation to changing diets. The hominid and murid lineages share PSGs involved in cell differentiation (*FZD2*) and reproduction (*TXNDC3*). The *TXDNC3* protein (Sptrx-2) participates in the final stages of sperm tail maturation in the testis and/or epididymis and is a structural component of the mature fibrous sheath of spermatozoa [2]. Proteins involved in reproduction tend to have evolved under positive selection [3, 4] and could be linked to speciation events.

We combined some of the common murid and hominid PSGs with PSGs only in the hominid or murid lineage. We then used this dataset to query the previously described database of biological interactions database to find significant networks and found three networks of genes involved in inflammation processes (Additional File 3). All of the functional processes concerned with inflammation are represented by the genes that appear in these networks: genes such as *F5*, *GP1BA*, *VWF*, *PTGIR* are involved in blood coagulation, cell-adhesion genes such as *MADCAM1*, *ITGAV*, genes that participate in inflammatory response (*TLR5, CXCL13, CCL19, CCL21*) and immune defence (*CD86, AZGP1*) as well as other related transport proteins. As the challenges to the immune system are constantly evolving and changing, we would expect immune system genes to be constantly under positive selection pressure to adapt to new incoming challenges. This can be seen with the MHC molecules [5] and may also be true of the genes in our network.

**Functional Classification of Chimpanzee PSGs**

A possible avenue to demystify the actuality of these adaptive chimpanzee genes is to ask whether these genes have any functional significance or biological grouping. Classification of PSGs using the PANTHER Biological Process (BP) ontology terms [6] showed that over a third (52 out of 162) of the chimpanzee PSGs are orthologues of human genes that are of unknown biological function. This proportion of 52 genes is significantly high (*p* < 0.036; Fisher’s exact test of proportions) compared to the number of genes with unknown function among the human PSGs (*p* < 0.053). Biological processes that were over-represented by chimpanzee PSGs included DNA repair, metabolism of cyclic nucleotides, peroxisome transport and the serine/threonine kinase signalling pathway.

**Taxon sampling does not affect detection of positive selection**

This study included five species exemplars whilst previous studies have been more restricted [7, 8]. However, the effect of taxon sampling on the detection of positive selection is largely unknown. To address this question we conducted permutation analyses of the original five-species alignments, to see if the exclusion of each non-human species in turn affects the results obtained.

For the first such set of analyses, after the sequence was removed from the alignment, the remaining sequences were not realigned prior to analysis with the branch-site model. For the second set of analyses, the sequences remaining after a sequence was excluded were realigned. The number of PSGs each analysis had in common with the five-species alignment was calculated.

In both realigned and non-realigned analyses, the effect of taxon elimination on the number of PSGs was most pronounced on the ancestral lineages, resulting in the most severe loss in the number of detected PSGs (Table 2). This could be because there are no direct observations to obtain data for the internal branches; instead the sequence is inferred by ancestral reconstruction. If more species were used in the analysis, sequence reconstruction can be performed more accurately, hence the removal of one sequence influences reconstruction and can considerably affect the number of positive genes detected.

Among the extant lineages, removal of the dog sequence seems to have had the most severe effect (percentages of overlapping genes range from 50-89%), probably because dog is an outgroup in the species phylogeny and hence removal of this branch substantially decreased the power to detect positive selection. The exclusion of a sequence from the remaining three taxa has slightly lesser effects (percentages range from 57% to 92%) but an assessment of the number of genes that were positive in all three analyses (Additional File 4) shows that detection of positive selection in a substantial portion of genes are robust to all manipulations and species exclusions.

The high numbers seen are a reflection of the stability of the results regardless of changes in the number of taxa used, changes in tree topology and also changes in the alignment. We conclude that once rigorous orthologues are established the results are fairly consistent regardless of the species being removed. We can conclude that PAML is robust to the effects of taxon sampling and the determination of PSGs reported in this study is accurate and not an effect of taxon sampling.

**Chimpanzee PSGs are lineage-specific**

To determine whether the adaptive genes seen in the chimpanzee were also under positive selection in other non-human primates, we performed a pilot study with other primate sequences, whose draft assemblies were available (macaque, orangutan and marmoset) at the same coverage (5-6x) as the chimpanzee genome. From the 162 chimpanzee PSGs, we selected 11 genes that had the most residues predicted to be under positive selection. We obtained orthologous sequences by gene prediction for these genes for the three primates and performed positive selection analyses under the branch-site model on the resulting new alignments. All primate branches and branches leading to primates were tested as the foreground lineage in turn.

All 11 genes remained subject to positive selection along the chimpanzee lineage (Table 3). Some of the genes were under positive selection along the other primate lineages, with five of the genes under positive selection in the marmoset lineage (a New World monkey).

The amino acid differences observed in the 11 chimpanzee sequences are specific to the chimpanzee, with the other primate sequences having the same state as the human sequence. This suggests that the human state is the ancestral state and the chimpanzee state is the derived change with the adaptation observed in the chimpanzee being lineage-specific.

**Figure Legends**

**Additional File 3: PSGs along the hominid and murid lineages cluster to form networks involved in inflammatory processes**

**Additional File 4: Summary of results from taxon exclusion studies**

Circle A. Five-species alignment

Circle B. Species exclusion and no realignment

Circle C. Species exclusion and realignment

The exclusion of the chimpanzee sequence and the mouse sequence are compared to the exclusion of the dog sequence, which as the outgroup of the tree used in the analyses had the most impact on the number of PSGs on the other lineages.

**Tables**

**Table 1. PSGs in adjacent lineages**

| **Lineages** | ***p* value** | **Gene Symbol** |
| --- | --- | --- |
| Human and Chimpanzee | *p* < 6.864e-10 | ABCF1, ALPPL2, CNGA4,, PIK3C2G, ZRSR2, KIAA0372, C8ORF42, ANGEL1, MICALCL, OR4F17, ZNF324B, ANKRD35, GIPC2, RUFY4, RBM16, MGC50722, INPP5B |
| Mouse and Rat | *p* < 4.651e-04 | SYT4, STS, CA6, CDC14B, TARP, RRAGA, DHDH, C19ORF16 |
| Hominid and Murid | *p* < 8.256e-05 | TXNDC3, ITGAV, MRC2, CLSTN2, ZNF665, CD86, FZD2, F5 |

The *p* value is from Fisher’s exact test of proportions to test for significance.

**Table 2. Number of PSGs after sequence exclusion**

| **Taxon removed** | **Human** | **Chimpanzee** | **Hominid** | **Mouse** | **Rat** | **Murid** | **Dog** | |
| --- | --- | --- | --- | --- | --- | --- | --- | --- |
| Number of PSGs after taxon exclusion and no realignment | | | | | | | | |
| Chimpanzee |  |  |  | 74 (88) | 87 (75) | 80 (70) | 113 (79) | |
| Dog | 54 (67) | 174 (89) |  | 67 (74) | 77 (58) |  |  | |
| Rat | 54 (83) | 162 (92) | 53 (57) |  |  |  | 89 (72) | |
| Mouse | 59 (80) | 163 (90) | 50 (54) |  |  |  | 79 (81) | |
| Number of PSGs after taxon exclusion followed by realignment | | | | | | | | |
| Chimpanzee |  |  |  | 72 (77) | 100 (70) | 72 (56) | | 123 (75) |
| Dog | 50 (50) | 200 (86) |  | 66 (65) | 87 (54) |  | |  |
| Rat | 48 (57) | 188 (86) | 58 (52) |  |  |  | | 98 (68) |
| Mouse | 62 (67) | 189 (86) | 46 (46) |  |  |  | | 92 (64) |

Numbers (*p* < 0.05) are only shown for lineages for which there were no changes in topology when the taxon in question was removed from the tree.

In parentheses are the number of PSGs that were common to the four-species analyses and the original five-species alignment (result set B), as a percentage of number of PSGs in the five-species alignment.

**Table 3.** **Test statistic (2x∆*ln*L) for 11 chimpanzee PSGs genes before and after analysis with other primate sequences.**

| **Gene** | **Chimpanzee (original analysis** | **Chimpanzee (after primate sequence addition)** | **Orangutan** | **Macaque** | **Marmoset** | **Ancestor to HCOM** |
| --- | --- | --- | --- | --- | --- | --- |
| AQP2 | 87.08** | 77.41** |  |  |  |  |
| EEF1G | 40.88** | 36.23** |  |  | 131.94** | 8.54* |
| ELF4 | 78.34** | 64.28** |  | 166.93** | 28.11** |  |
| HCRTR1 | 49.61** | 53.64** |  |  |  |  |
| TKTL1 | 25.85** | 24.84** |  |  | 19.74** |  |
| DYRK2 | 78.86** | 85.44** |  |  | 8.74* |  |
| PIGV | 127.93** | 134.6** |  |  |  |  |
| PSD2 | 150.79** | 139.6** | 216.47** |  | 66.84** |  |
| CCDC97 | 90.45** | 100.5** |  |  |  |  |
| CXorf38 | 52.64** | 57.87** |  |  |  |  |
| GIYD1 | 47.88** | 33.88** |  |  |  |  |

* *p* < 0.05; ** *p* < 0.001

**References**

1. Gilad Y, Man O, Glusman G: **A comparison of the human and chimpanzee olfactory receptor gene repertoires.** *Genome Res* 2005, 15(2):224-230.

2. Miranda-Vizuete A, Sadek CM, Jimenez A, Krause WJ, Sutovsky P, Oko R: **The mammalian testis-specific thioredoxin system.** *Antioxid Redox Signal* 2004, 6(1):25-40.

3. Wyckoff GJ, Wang W, Wu CI: **Rapid evolution of male reproductive genes in the descent of man.** *Nature* 2000, 403(6767):304-309.

4. Swanson WJ, Vacquier VD: **The rapid evolution of reproductive proteins.** *Nat Rev Genet* 2002, 3(2):137-144.

5. Bernatchez L, Landry C: **MHC studies in nonmodel vertebrates: what have we learned about natural selection in 15 years?** *J Evol Biol* 2003, 16(3):363-377.

6. Thomas PD, Kejariwal A, Campbell MJ, Mi H, Diemer K, Guo N, Ladunga I, Ulitsky-Lazareva B, Muruganujan A, Rabkin S *et al*: **PANTHER: a browsable database of gene products organized by biological function, using curated protein family and subfamily classification.** *Nucleic Acids Res* 2003, 31(1):334-341.

7. Clark AG, Glanowski S, Nielsen R, Thomas PD, Kejariwal A, Todd MA, Tanenbaum DM, Civello D, Lu F, Murphy B *et al*: **Inferring nonneutral evolution from human-chimp-mouse orthologous gene trios.** *Science* 2003, 302(5652):1960-1963.

8.Bakewell MA, Shi P, Zhang J: **More genes underwent positive selection in chimpanzee evolution than in human evolution.** *PNAS* 2007, 104(18):7489-7494.
