# Supplementary material for: The role of positive selection in determining the molecular cause of species differences in disease
Source: BMC Evol Biol. 2008 Oct 6;8:273. doi: 10.1186/1471-2148-8-273 (PMC2576240; doi:10.1186/1471-2148-8-273)
Supplement: Additional file 3 — PSGs along the hominid and murid lineages cluster to form networks involved in inflammatory processes. Network diagrams of positively selected hominid and murid genes that interact together and are involved in inflammatory functions. [file 1471-2148-8-273-S3.ppt]

## Slide 1
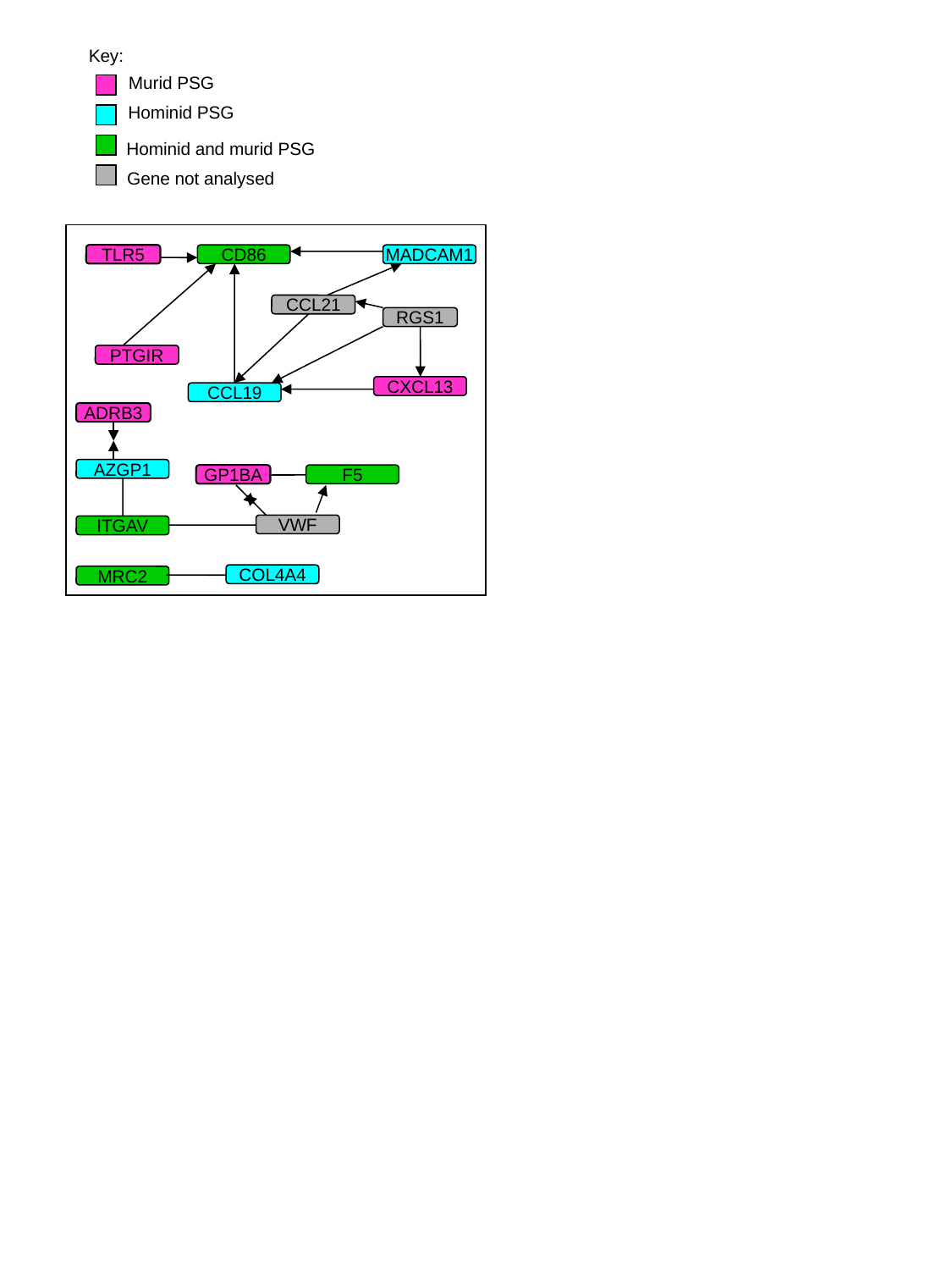

Key:
Murid PSG
Hominid PSG
 Hominid and murid PSG
Gene not analysed
TLR5
CD86
MADCAM1
CCL21
RGS1
PTGIR
CXCL13
CCL19
ADRB3
AZGP1
GP1BA
F5
VWF
ITGAV
COL4A4
MRC2
