# Supplementary material for: The role of positive selection in determining the molecular cause of species differences in disease
Source: BMC Evol Biol. 2008 Oct 6;8:273. doi: 10.1186/1471-2148-8-273 (PMC2576240; doi:10.1186/1471-2148-8-273)
Supplement: Additional file 4 — Summary of results from taxon exclusion studies. Circle representation of genes significant in one or more of the permutation studies. [file 1471-2148-8-273-S4.ppt]

## Slide 1
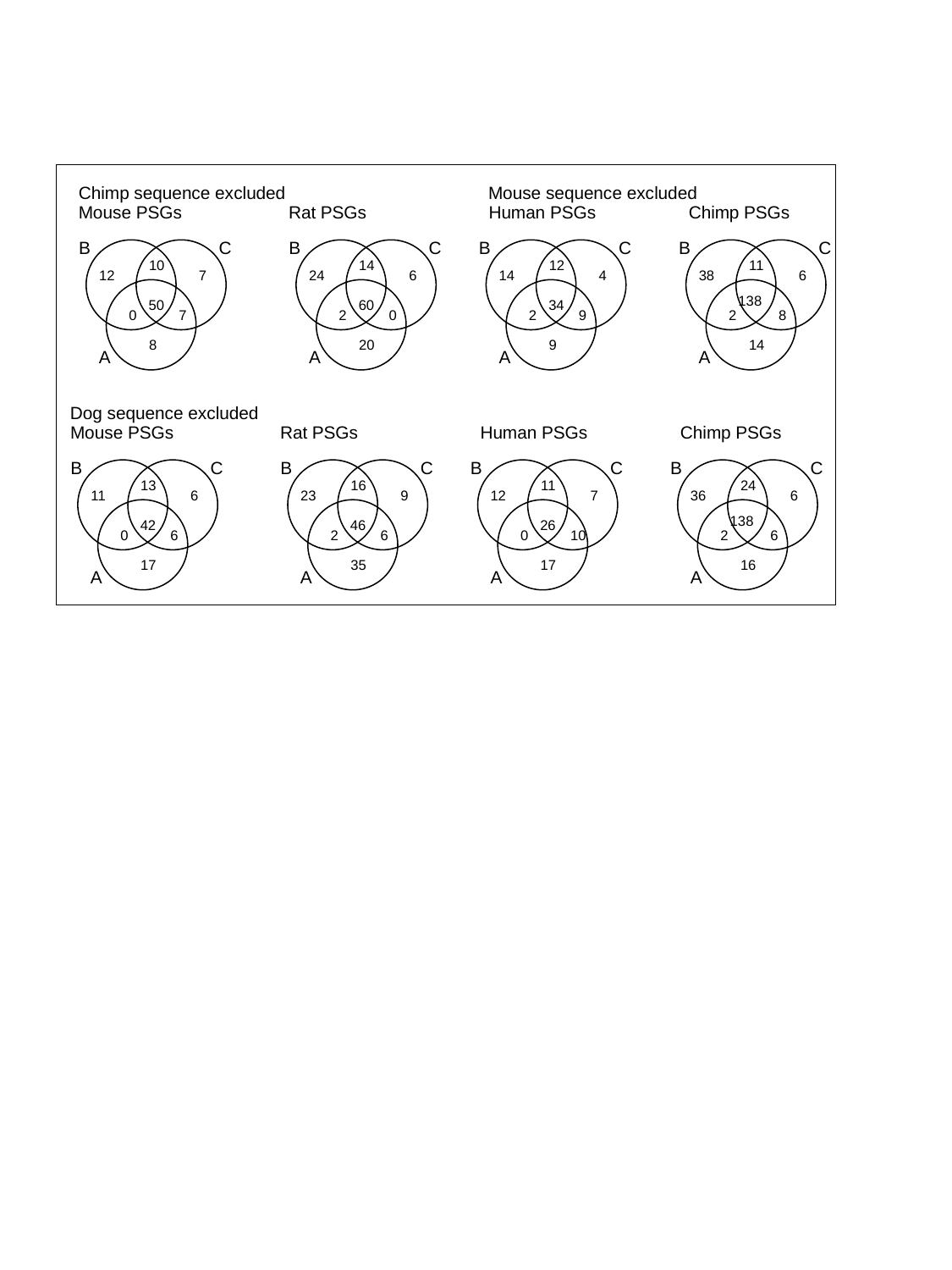

Chimp sequence excluded
Mouse sequence excluded
Human PSGs
Chimp PSGs
Mouse PSGs
Rat PSGs
B
C
10
12
7
50
0
7
8
A
B
C
B
C
12
14
4
34
2
9
9
A
B
C
14
11
24
6
38
6
138
60
2
0
2
8
20
14
A
A
Dog sequence excluded
Human PSGs
Chimp PSGs
Mouse PSGs
Rat PSGs
B
C
13
11
6
42
0
6
17
A
B
C
B
C
11
12
7
26
0
10
17
A
B
C
16
24
23
9
36
6
138
46
2
6
2
6
35
16
A
A
